# Practical Workflow for Cardiovascular Assessment and Follow-Up in Kawasaki Disease Based on Expert Opinion

**DOI:** 10.3389/fped.2022.873421

**Published:** 2022-06-09

**Authors:** Diana van Stijn, R. Nils Planken, Maarten Groenink, Nico Blom, Robbert J. de Winter, Taco Kuijpers, Irene Kuipers

**Affiliations:** ^1^Department of Pediatric Immunology, Rheumatology and Infectious Diseases, Emma Children's Hospital, Amsterdam University Medical Center (UMC), University of Amsterdam, Amsterdam, Netherlands; ^2^Department of Radiology and Nuclear Medicine, Amsterdam University Medical Center (UMC), University of Amsterdam, Amsterdam, Netherlands; ^3^Department of Cardiology, Amsterdam University Medical Center (UMC), University of Amsterdam, Amsterdam, Netherlands; ^4^Department of Pediatric Cardiology, Emma Children's Hospital, Amsterdam University Medical Center (UMC), University of Amsterdam, Amsterdam, Netherlands

**Keywords:** Kawasaki disease, mucocutaneous lymph node syndrome, imaging, coronary artery aneurysms, cardiovascular assessment

## Abstract

**Background:**

Approximately 25% of the patients with a history of Kawasaki disease (KD) develop coronary artery pathology if left untreated, with coronary artery aneurysms (CAA) as an early hallmark. Depending on the severity of CAAs, these patients are at risk of myocardial ischemia, infarction and sudden death. In order to reduce cardiac complications it is crucial to accurately identify patients with coronary artery pathology by an integrated cardiovascular program, tailored to the severity of the existing coronary artery pathology.

**Methods:**

The development of this practical workflow for the cardiovascular assessment of KD patients involve expert opinions of pediatric cardiologists, infectious disease specialists and radiology experts with clinical experience in a tertiary KD reference center of more than 1000 KD patients. Literature was analyzed and an overview of the currently most used guidelines is given.

**Conclusions:**

We present a patient-specific step-by-step, integrated cardiovascular follow-up approach based on expert opinion of a multidisciplinary panel with expertise in KD.

## Introduction

Kawasaki disease (KD) is a pediatric systemic vasculitis of unknown etiology, which mainly affects children under the age of 5 years. KD is generally a self-limiting acute inflammatory disease that predominantly affects the coronary arteries. Inflammation of the coronary arteries may lead to coronary artery aneurysms (CAAs) which can lead to adverse cardiac complications. CAAs develop in approximately 25% of untreated patients and can be reduced to 9% if treated timely (1–4). Some risk factors have been identified for the development of CAAs such as: resistance to treatment, delayed treatment (later than 10 days after fever onset), male gender, incomplete KD and an age at the end-spectrum of the classical age for KD (5, 6). Studies have shown that CAA regression mainly occurs within the first 2 years after onset of disease (7). Regression seems to occur with a predilection for some conditions: when aneurysms do not show calcification, have a smaller diameter, or have an ectatic shape (1).

Pediatric cardiologists use echocardiography to diagnose and monitor KD patients. Currently *Z* scores are most commonly used for risk assessment and clinical decision making whereas in the past, luminal diameters were used. CAAs can be classified according to their *Z* score; small aneurysms: ≥ 2.5 <5.0, medium aneurysms: ≥ 5.0 <10.0, large/giant aneurysms: ≥ 10.0. Patients in the last category and even more so in case of a *Z* score ≥ 20.0, are at the highest risk for the development of stenosis and formation of coronary thrombus, which may lead to myocardial ischemia, infarction and sudden death (8). Multiple *Z* score calculators are used and inter-variability in *Z* score calculation has been reported, especially larger dimensions of the coronary arteries show larger discrepancies between different calculators (9). These discrepancies between *Z* score systems can lead to variation in diagnosis and management (10), more research is required to identify the ideal *Z* scoring system.

CAAs in the right coronary artery (RCA), in the left anterior descending coronary artery (LAD), in the circumflex (Cx) and CAAs with complicated architecture also have an increased risk for luminal narrowing and thrombosis. In previous reports perivascular brightness was considered an early sign for CAA formation (11), but more recent studies showed that perivascular brightness and lack of tapering were non-specific findings that could also be found in healthy children and children with fever without KD (12, 13).

While striving toward a uniform and realistic monitoring schedule for KD patients, we had previously set up a practical workflow at our center (14). Since our previous report, we obtained experience with state-of-the art coronary CT angiography (cCTA) (15, 16), which can assess the coronary artery tree at great detail with reduced radiation exposure. Therefore, cCTA is now fully integrated into our updated cardiovascular follow-up workflow, to avoid underreporting CAAs and prevent cardiac ischemia. This update gives an overview of the most current guidelines with practical recommendations based on the clinical experience from our single center in over 1,000 KD patients.

### Echocardiography

#### Coronary Artery Pathology

**Figure 1:**
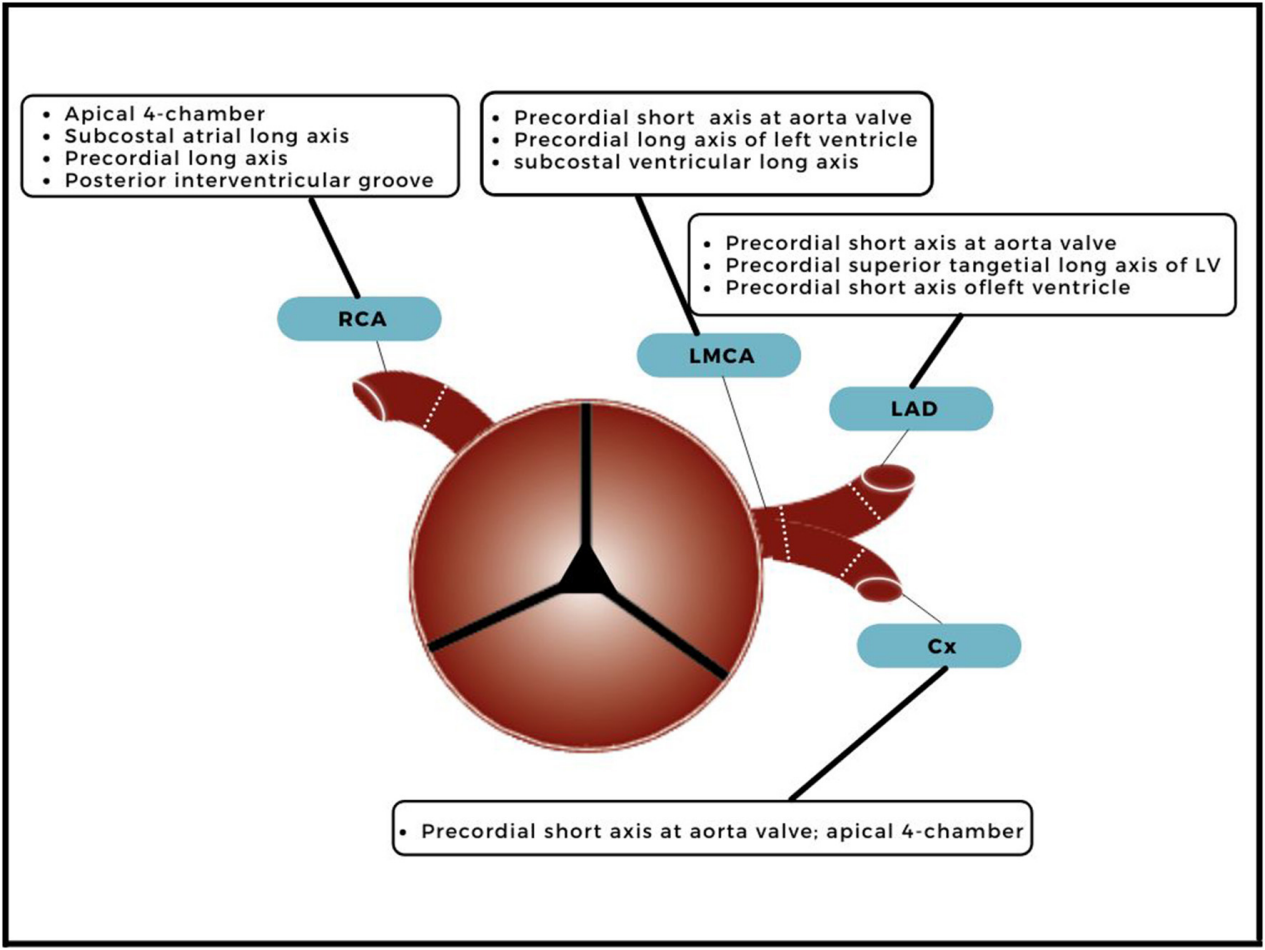
Echocardiographic view and measuring points. A transverse plane of the ascending aorta, just above the aortic valve, with branching of the right coronary artery (RCA), left main coronary artery (LMCA), left anterior descending artery (LAD) and circumflex (Cx). The dotted lines indicate the measuring points and echocardiographic views for best visualization measurement are included.

Echocardiography is the cornerstone of the acute and long-term cardiovascular assessment in KD patients during adolescence. When visualizing the coronary arteries, it is important at which location the luminal diameters are measured (Figure 1); after which these diameters are calculated to *Z* scores with the Body Surface Area (BSA). *Z* scores are acquired by echocardiography and not validated for other imaging modalities. Each coronary artery has a different echocardiographic view for best visualization (Figure 1). Echocardiography is limited in visualizing the distal sections of the coronary artery tree due to limited ultrasound windows, and diagnostic accuracy has been questioned (9, 15). Therefore, complementary imaging modalities should be considered. However, there is minimal risk for distal involvement without proximal involvement (15, 17).

#### Non-coronary Cardiac Involvement

Cardiac manifestations in KD can occur independently of coronary artery lesions. Therefore, not only coronary artery assessment but also echocardiographic assessment of the cardiac chamber size and function in the acute phase is necessary. The inflammatory process in KD can affect the pericardium, myocardium, endocardium and the valves. (Peri) myocardial inflammation occurs allegedly in all KD patients and very likely even before the development of coronary arteritis (18). It can cause transient cardiac dysfunction, often described as inflammatory myocarditis, but only a minority of patients exhibit heart failure symptoms (19, 20). Echocardiographic findings indicating perimyocardial inflammation in the acute stage of the disease are: decreased left ventricular fractional shortening or ejection fraction, mitral valve regurgitation, and pericardial effusion (21, 22). Myocardial inflammation is known to mainly cause edema without permanent cell damage (18). Apart from the acute phase, echocardiography continues to be of additional value during follow-up to monitor potential evolving complications such as reduced ventricular wall movements as signs of ischemia or (missed) infarction. Aneurysms of the aortic root (*Z* score ≥ 2) are present in 10% of KD patients (23), which does not seem to regress 1 year after onset of disease (24). Long-term surveillance is necessary to determine the importance of this finding, which is unclear to date. Aortic dissection has not been reported in KD patients thus far.

#### Electrocardiography

Electrocardiography is generally used to exclude myocardial ischemia and infarction. Atrioventricular (AV) block, repolarization abnormalities and arrhythmias may occur as a result of the inflammation as well. Due to stenotic or thrombotic CAAs, KD patients can exhibit abnormal electrocardiography, indicating ischemia or myocardial infarction. Such signs must have immediate dollow-up imaging and biochemical check in plasma for cardiac enzymes.

### Coronary Artery Angiography

Initially, coronary artery angiography (CAG) has been advocated to be performed regularly depending on the size of CAA or strongly suspected risk of ischemia. CAG is still considered the “gold standard”, if necessary complemented with intracoronary imaging. Several adverse cardiac events haven been described when CAG is performed in the acute phase of KD (25). Furthermore, patients are exposed to a relatively high radiation dose and in children the procedure needs to be performed under general anesthesia. This has caused clinicians to consider alternative imaging techniques. Studies have shown that the cCTA, compared to CAG is reliable and useful in the complete visualization of the coronary arteries and accurate measurement of possible CAAs (26). Invasive angiography should only be considered when revascularization by either interventional approach or surgery is indicated based on non-invasive imaging.

### Coronary Computed Tomographic Angiography

cCTA is a non-invasive anatomical imaging modality that is ideal for overall detailed 3D coronary artery assessment. It can detect aneurysms, stenosis, thrombosis and calcification at a much greater detail than echocardiography (15). Echocardiography can miss pathology in the distal segments of the coronary artery tree due to a limited ultrasound window, whereas cCTA does not have that limitation and gives a total overview. Moreover, and indicated in our previous study, the circumflex (Cx) most often cannot be detected by echocardiography, but is properly visualized in all patients by cCTA and may contain large CAAs as well (15). Motion artifacts are infrequent in cCTA imaging but may occur (16).

In the past, radiation exposure has been a limiting factor for the application of cCTA in pediatric patients. State-of-the-art CT scanners enable imaging of the coronary artery tree at acceptable radiation exposure and is generally considered an alternative to CAG, as non-invasive anatomical imaging modality in KD (15). Between 1996–2010 a mean radiation exposure acquired by chest CT in children <5 years, 5–9 years and 10–14 years has been 5.3 mSv, 7.5 mSv, 6.4 mSv, respectively (27). By optimization, radiation exposure can be reduced, substantially lowering the risk of radiation induced cancers (27). With a third generation dual-source CT scanner we reached a median effective dose (ED) of 1.5 mSv in KD patients for the evaluation of the coronary arteries (15), while natural background radiation has been estimated at 3 mSv per year for adults and a chest X-ray at 0.01 mSv (28). Apart from the actual exposure dose per procedure, the exposure is also determined by the heart rate. At irregular and high heart rates the acquisition window is widened resulting in a higher radiation dose. Furthermore, a higher heart rate is associated with more motion artifacts.

To reduce motion artifacts, if appropriate, beta-blockers should be prescribed if the heart rate exceeds 72 beats per min. When using a dual-source scanner, good quality acquisitions at higher heart-rates are possible by applying a prospective ECG-triggered sequential scan, instead of a high-pitch spiral scan. To avoid motion artifacts, children between the age of 18 months and 4 years are scanned under general anesthesia. Children younger than 18 months are scanned with a “feed and wrap” method, where the child is first fed then swaddled to induce natural sleep during scanning.

If patients are to transition to the adult cardiologist, cCTA in the late adolescence can give a starting point for further follow-up.

### Cardiac Magnetic Resonance Imaging

CMR is a non-invasive and radiation free functional imaging modality. Before the arrival of the dual-source CT scanners, CMR was the preferred imaging method for the coronary artery assessment in addition to echocardiography. However, studies have shown that CMR is not as accurate as the cCTA for the detection of CAAs, thrombosis, calcification and stenosis (16). Nevertheless, CMR does offer the evaluation of the cardiac function, volumes and myocardial perfusion with pharmacological stress testing which is essential to assess reversible ischemia and visualization of myocardial scarring with delayed contrast enhancement (29–31).

The disadvantage of MRI is that imaging is more sensitive to motion artifacts due to the long scan time that is required to acquire all the proper image sequences. Involuntary subject motion and cardio/respiratory motions including high and variable heart rate, can significantly affect image quality. Children under the age of +/- 10 years (depending on compliance) often require anesthesia because they are unable to stay motionless and compliant for the duration that the CMR requires to obtain high-quality images.

### Stress Testing for Inducible Myocardial Ischemia

Echocardiography, MRI or single-photon emission computed tomography (SPECT) stress testing is a noninvasive procedure that can detect inducible ischemia in KD patients. In general, exercise stress is preferred over pharmacologically induced stress (32); therefore, the success rate depends highly on the ability of the child to cooperate. Stress testing does not have a fixed place in our practical workflow but is performed on indication (when thrombosis and/or stenosis is suspected, when having clinical symptoms such as chest pain, ECG changes and/or severe abnormalities on previous imaging). Little research has been performed thus far to determine the clinical benefits (treatment and management) of stress testing in addition to CMR and/or CT.

### Future Imaging Perspectives: Hemodynamics

Not only the *Z* score of the CAA matters for risk stratification, also shape, number of CAAs should be examined because they influence hemodynamics. Hemodynamics are relevant in the development of thrombosis. Insight into the changes in blood flow and shear stress might be useful in developing a more specific risk assessment for patients with aneurysms. Invasive studies (doppler flow wire measurements) suggest that stagnation of flow and low shear stress is associated with the risk of thrombus formation (33, 34). Image-based modeling to quantify hemodynamics and shear stress showed that hemodynamic parameters can identify aneurysms at risk for thrombotic lesions (35, 36).

**Figure 2:**
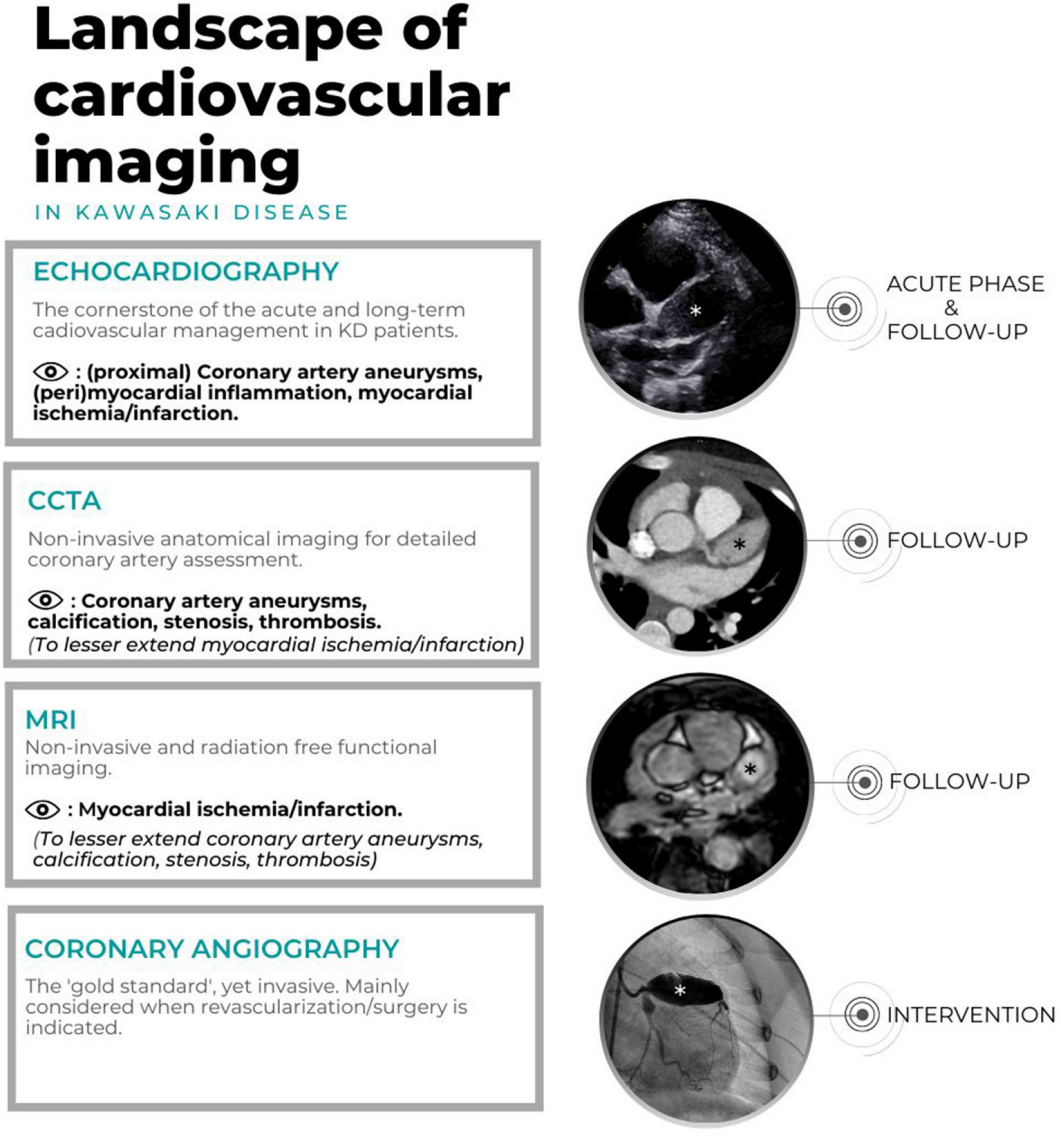
Landscape of cardiovascular imaging in KD. Echocardiography, CAG, cCTA of one patient with giant CAA in the LAD (*) and CMR of another patients with giant CAA in LAD (*).

Echocardiography, cCTA, CMR and CAG are of importance for the risk assessment of aneurysmatic lesions, to further optimize anticoagulant treatment and assess the timing of coronary artery bypass grafting or percutaneous transluminal coronary angioplasty (PTCA). Figure 2 shows the landscape of cardiovascular imaging modalities in KD patients.

### Assessment of Guidelines

#### Acute, Subacute and Convalescent Phase Until 3 Months After Onset of Disease

##### American Heart Association

The American Heart Association (AHA) guidelines (17) recommend echocardiography as the first choice imaging modality, the frequency depends on the presence of CAA, stability and size (Table 1). ECG is not routinely adopted in the AHA cardiovascular assessment guidelines of the acute phase.

**Table 1:** Summary of AHA guidelines echocardiography during acute and subacute an convalescent phase (<3 months).

	**Detection of**	**Interval echocardiography**	**Other**
Uncomplicated patients	CAA	At diagnosis, 1–2 weeks after treatment, 4–6 weeks after treatment	
Z score > 2.5	CAA	At least 2x per week until progression stopped	
If expanding or giant CAA	Coronary artery thrombosis	−1x per week in 1^st^ 45 days - 2x per week while expanding, otherwise: monthly until 3^rd^ month	In case of giant CAA consider cCTA/CMR/CAG at baseline (within 2–6 months)

##### Japanese Circulation Society

Interestingly, the guidelines of the Japanese Circulation Society (JCS) (37) do not define a CAA in the first month of disease, only if the lesions persists after 1 month of disease. If the CAA, independent of its size, resolves within 1 month it will be classified as a transient dilation. KD patients categorized with no dilation or transient dilation are recommended to have an ECG and echocardiogram at 1 and 2 months. For the patients with a remaining CAA, with a stenotic lesion confirmed by CAG (with or without ischemia), the JCS recommends to consider cCTA, CMR or CAG in the convalescent phase. The JCS does not have step-by-step recommendations for the acute phase (i.e., the first 3 months) other than the last mentioned.

#### Long-Term Follow-Up (From 3 Months Onward)

Both guidelines propose their long-term cardiovascular assessment based on the findings by echocardiography during the first 3 months. Both guidelines have recommendations for regressed CAAs over time, for the long-term follow-up. The long-term cardiovascular assessment of the AHA and JCS are summarized in Tables 2, 3, respectively.

**Table 2:** Summary of AHA guidelines for the long-term cardiovascular assessment (> 3 months).

**Classification**	**Interval echocardiography + ECG**	**cCTA, CMR, CAG**	**Inducible myocardial ischemia (stress echocardiography MRI, stress nuclear medicine, positron emission tomography (PET))**
No Dilation and dilation	Consider: up to 12 months*		
Regression small CAA to normal/dilation	Every 1–3 years, not performing routine echocardiography may be considered unless patient has symptoms or signs of ventricular dysfunction/myocardial ischemia	Consider: if inducible ischemia/ventricular dysfunction	Consider: every 3–5 years or if patient has symptoms**
Regression medium CAA to small CAA	Yearly	Consider: 3–5 years	Every 2–3 years or if patient has symptoms**
Regression medium CAA to normal/dilation	Every 1–2 years, not performing routine echocardiography may be considered unless patient has symptoms or signs of ventricular dysfunction/myocardial ischemia	Consider: if inducible ischemia	Every 2–5 years if patient has symptoms**
Regression giant CAA to medium CAA	Every 6–12 months	Consider: 2–5 years	Every year if patient has symptoms**
Regression giant CAA to small CAA	Every 6–12 months	Consider: 2–5 years	Every 1–2 years or if patient has symptoms**
Regression giant CAA to normal/dilation	Every 1–2 years, not performing routine echocardiography may be considered unless patient has symptoms or signs of ventricular dysfunction	Consider: 2–5 years	Every 2–5 years or if the patient has symptoms**
Remaining small CAA	6 months, 1 year, every year onward is reasonable	Consider: 3–5 years	Every 2–3 years or if patient has symptoms**
Remaining medium CAA	3 months, 6 months, 1 year. Every 6–12 months onward is reasonable	Consider: 2–5 years	Every 1–3 years or if patient has symptoms**
Remaining giant CAA	6, 9, 12 months in 1^st^ year and every 3–6 months onward	Consider: Baseline within 2–6 months or in 1^st^ year, consider every 1–5 years onward	Every 6–12 months if patient has symptoms**

*Dilation is a CAA with Z score ≥2– <2.5*.

*Ongoing follow-up to 12 months may be considered, if dilation is persistent after 4–6 weeks then it is reasonable to continue follow up to 12 months or even every 2–5 years.

**Suggestive for ischemia or signs of ventricular dysfunction.

**Table 3:** Summary of JCS guidelines for the long-term cardiovascular assessment (> 3 months).

**Classification**	**Interval echocardiography + ECG**	**cCTA, CMR, CAG**
No Dilation and transient dilation*	6, 12 months and 5 years (or yearly) until 5 years old	
Regression small CAA (normalization)	Yearly	Consider: 1 year/when CAA regresses, recommended when finishing high school**
Regression medium/giant CAA (normalization)	Every 6–12 months	Consider: 1 year then 3–5 years**
Remaining small CAA	Yearly	Consider: 1 year then 3–5 years, desirable to perform CAG at least once***
Remaining medium CAA	Every 6–12 months	Consider: 1 year then 2–5 years, desirable to perform CAG at least once ***
Remaining giant CAA	Every 6–12 months	Consider: 1 year then 1–5 years, desirable to perform CAG at least once ***
Coronary artery stenotic lesion + ischemia	Consider timely	Consider timely
Coronary artery stenotic lesion	Every 6–12 months	Consider: 1 year then 1–5 years

*Transient dilation is defined as any CAA in the first month.

^**^Periodic check-ups are advised due to the potential progression with calcification or stenosis after 10–20 years.

^***^According to the JCS, it is desirable to perform CAG at least once in patients with dilation and CAAs because of discrepancies between echocardiography and CAG.

The Japanese guidelines consider additional imaging modalities (cCTA, CMR or CAG). The JCS mentions that stress testing is important for myocardial ischemia detection. Stress echocardiography or CMR using either pharmacological stress or exercise could be valuable in addition to the exercise ECG. The JCS describes “periodic check-ups” in regressed medium and giant CAAs, but it is unclear what these check-ups should encompass as a minimum. In addition, the JCS mentions that it is desirable to perform CAG at least once in patients with coronary artery dilation, due to discrepancies between echocardiography and CAG.

## Cardiovascular Assessment: Actionable Workflow Based on Expert Opinion From a Tertiary KD Clinic

### Acute, Subacute and Convalescent Phase (Up to 3 Months)

**Figure 3:**
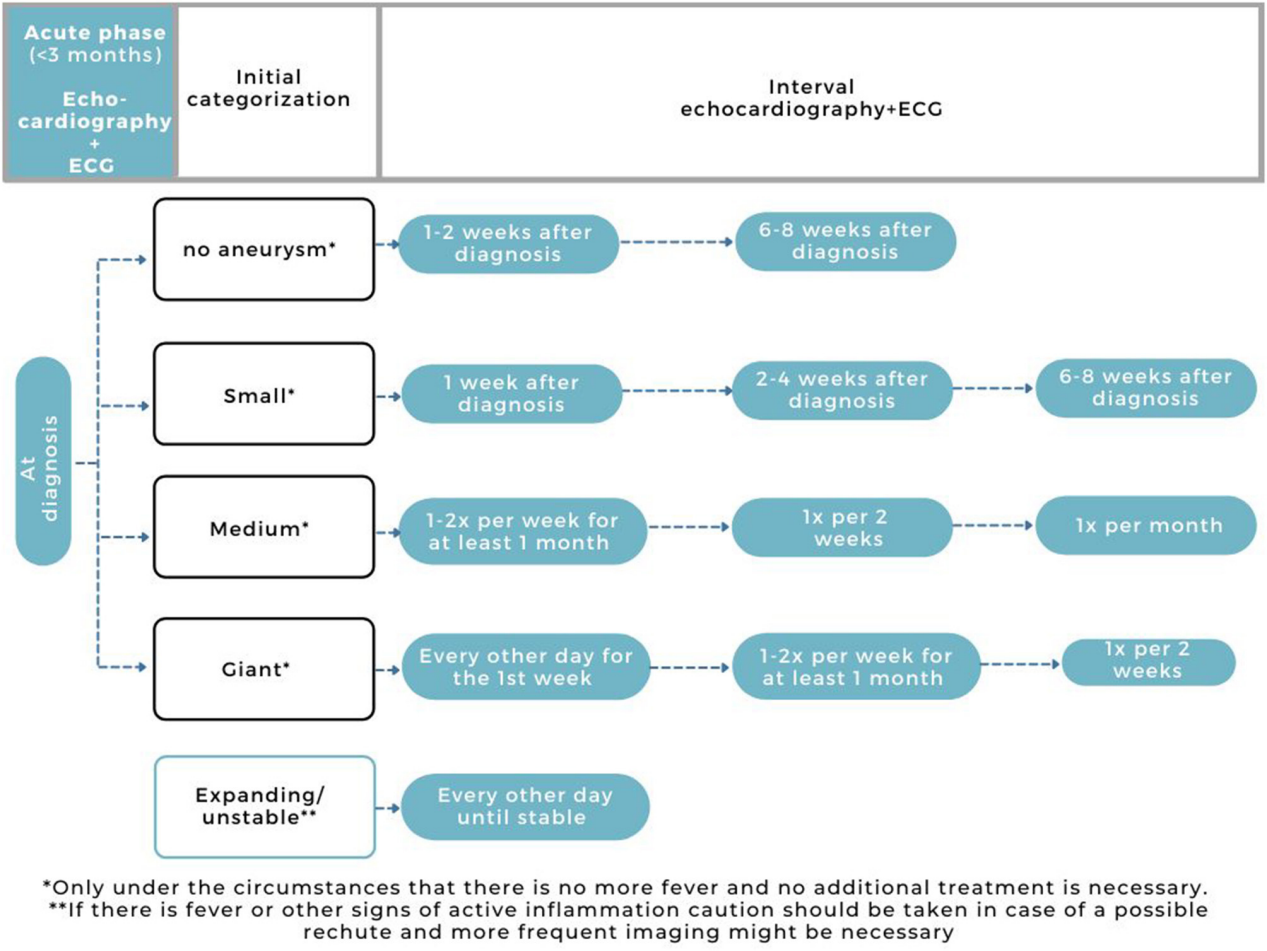
Current practice for cardiovascular assessment in the acute phase of KD (<3 months).

Based on our experience and more or less similar to the AHA, we apply echocardiography in uncomplicated patients in the acute phase: at diagnosis, week 1-2, and week 6-8 (Figure 3). In patients with expanding/unstable CAAs upon the first echocardiographies, or with signs of ongoing inflammation (fever or persistent/slowly decreasing CRP), we strongly recommend more frequent echocardiographic imaging (Figure 3). When echocardiography results are indefinite and/or more complications are suspected, additional imaging can be of additional value, also in the acute phase.

## Long-Term Follow-Up

**Figure 4:**
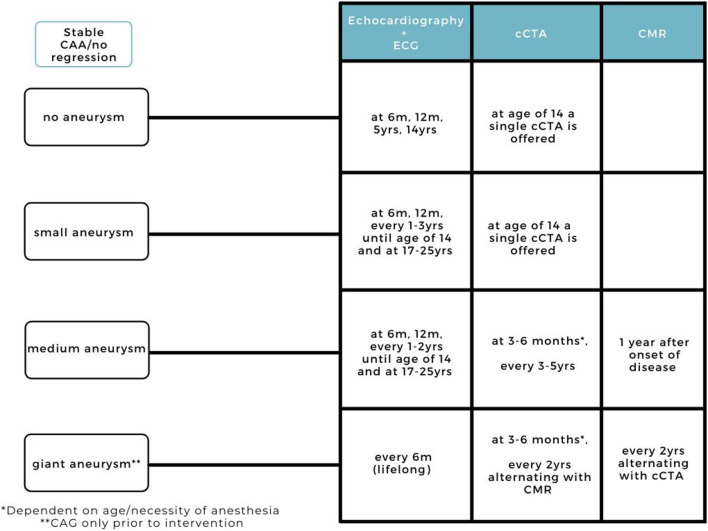
Current practice for long-term cardiovascular follow-up (>3 months) in KD patients (stable CAAs, without regression).

**Figure 5:**
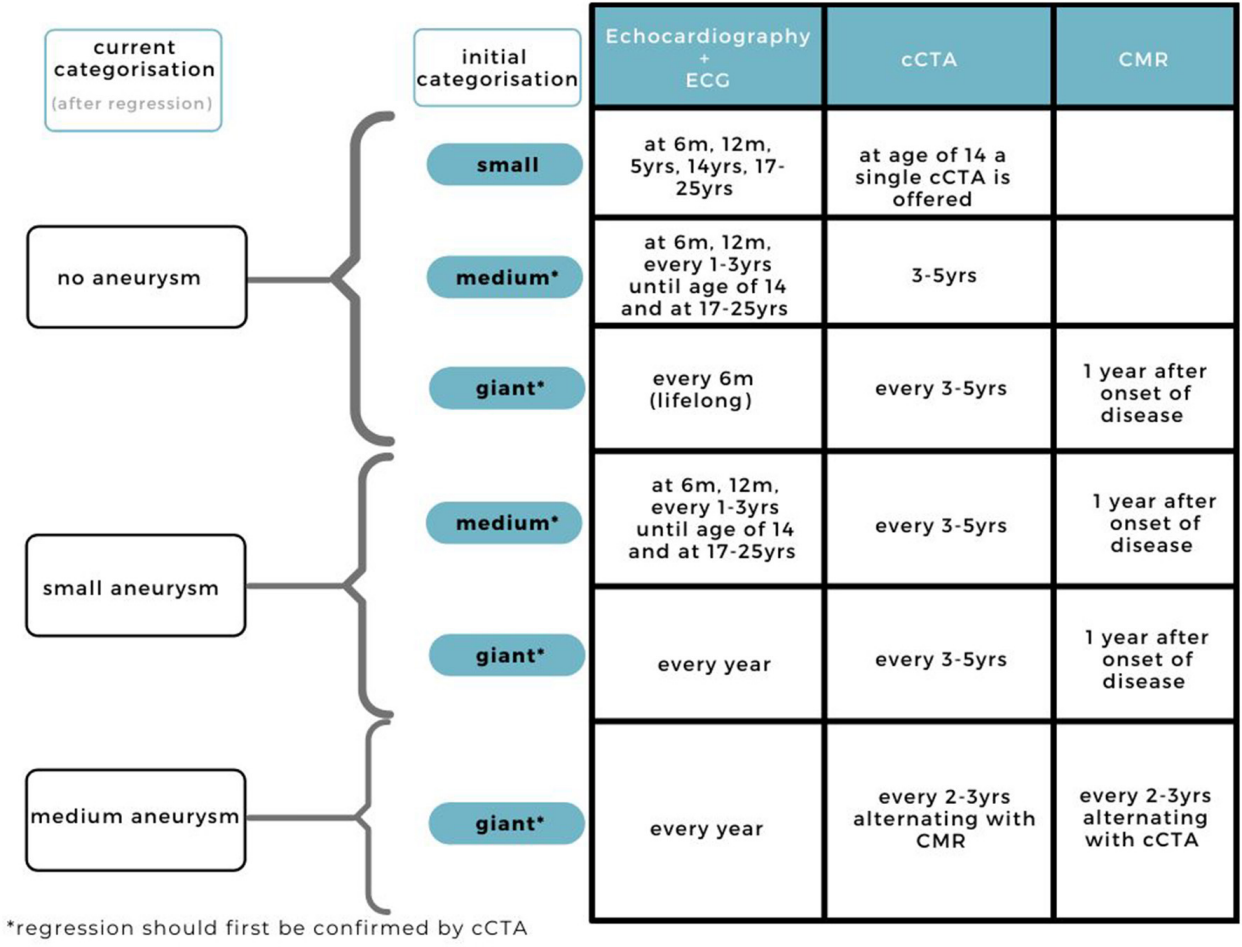
Current practice for cardiovascular assessment during follow-up in KD patients with regressed CAAs.

Our current practice for the long-term follow-up is divided in a stable situation (Figure 4) and one of regression of the aneurysmatic lesion (Figure 5).

### Stable Situation

#### No Aneurysm

Similar to AHA and JCS guidelines for KD patients with no coronary artery involvement on echocardiography, we do not recommend any additional imaging methods. We follow a different frequency of echocardiography, encompassing a follow-up at 6 months, 12 months, 5 years, and at 14 years (Figure 4).

At the final check we offer an extensive risk factor evaluation for the detection of additional risk factors on top of KD (including lipid profile, familial cardiovascular disease and atherosclerosis risk interpretation). We combine lipid profiles with cCTA for plaque, CAA and thrombosis detection (Figure 4). When repeatedly abnormal lipid profiles are detected (increased LDLc, total cholesterol, LpA), we perform a targeted next-generation sequencing panel of 30 genes to exclude familial dyslipidemia). If abnormal, life style advice and cholesterol lowering medication is provided with patient-tailored follow-up.

#### Small Aneurysm (Z Score ≥ 2.5 <5.0)

At our center, we offer patients with small aneurysms echocardiographic follow-up combined with ECG similar to the AHA guidelines, at 6 months, 12 months, and every 1–3 years until the age of 14 where we combine imaging with extensive assessment of risk factors, essentially as mentioned above. Based on current experience with state-of-the-art cCTA, we recommend additional imaging in KD patients with coronary artery involvement. By detecting missed aneurysms, a more accurate CAA classification can be established and possible complications due to under-treatment can be prevented. We suggest this additional imaging at the age of 14 to avoid motion artifacts and higher radiation exposure.

Depending on the imaging results, and additional (chemical) laboratory findings, transition to adult care is being discussed with the patients (and their parents) around this time (Figure 4). We combine lipid profiles with cCTA for plaque, CAA and thrombosis detection. Between the age of 17 and 25 we offer an extra visit if additional risk factors are present (blood tests, adipositas, familial cardiovascular disease).

#### Medium Aneurysm (Z Score ≥ 5.0 <10.0)

At our center, patients with medium aneurysms are offered more frequent echocardiographic follow-up than the AHA guidelines, but less frequent than the JCS guidelines. For additional imaging we have a clear schedule in which cCTA and CMR both have a distinct role and place in time. The first cCTA for the coronary artery assessment is performed at 3–6 months (depending on the age and necessity of anesthesia) and every 3–5 years (Figure 4). We do not perform additional imaging immediately after diagnosis, as the first weeks are known for diameter changes of the aneurysms also the patient can be more agitated/with tachycardia in the acute phase which can be challenging for high quality imaging therefore we wait until the 3rd month. We suggest to perform CMR 1 year after onset of disease to evaluate the cardiac function, volumes and fibrosis.

#### Giant Aneurysm Z Score ≥ 10

Patients with a CAA classification of a giant CAA (and even more so patients with a *Z* score > 20), have a risk of luminal narrowing, formation of thrombosis and major adverse cardiovascular events especially in patients with a CAA in the LAD and RCA (8). Therefore, we suggest to perform additional cardiovascular assessment at 3–6 months and thereafter every year, alternating CMR for cardiac function analysis and ischemia detection, and cCTA for assessment of the coronary artery tree and evaluation of diameter, calcification, formation of thrombosis, plaque deposition, and arterial stenosis (Figure 4), together with lipid profiles as mentioned above, including repeated monitoring for blood parameters because of the double (or triple) anticoagulant medication.

### Regression

When regression is suggested upon echocardiography, and confirmed by cCTA, we suggest an adjusted follow-up routine depending on the change in *Z* score classification (Figure 5). Giant and medium aneurysms that have regressed are at risk for stenosis, therefore frequent imaging, especially during the regression, is advised. Depending on the stability of the regressed CAA and parents, it may be decided to deviate from this imaging frequency based on the doctor's own discretion.

## Discussion

Based on our experience in practice, combined with the current guidelines, we have presented our patient-specific follow-up workflow for the cardiovascular assessment in KD patients.

The first 3 months since the onset of disease is a precarious time and the frequency of echocardiography is dependent on a few factors: presence or absence of fever, expanding/unstable or stable luminal diameter of the coronary arteries and the *Z* score category in case of an aneurysm. Especially patients with ongoing inflammation such as IVIG resistance rechute or persistent fever are at risk for complications. Long-term follow-up is dependent on the initial CAA classification and extent of regression.

## Conclusion

Based on our experience in a tertiary single center KD center, and the current guidelines we have presented our cardiovascular assessment flowchart during the acute phase and long-term follow-up. With the current acceptable radiation exposures, cCTA now plays a significant role in patients positive for coronary artery involvement and CMR for cardiac function and infarction size.

## Author Contributions

DS conceptualized the study and drafted the initial manuscript. TK and IK contributed equally as co-senior authors and conceptualized the study, coordinated, supervised and reviewed the manuscript for important intellectual content, and revised the manuscript. RP, MG, NB, and RW conceptualized the study and reviewed for important intellectual content and revised the manuscript. All authors approved the final manuscript as submitted and agree to be accountable for all aspects of the work.

## Funding

Funding was made available by the foundation “Kind en Handicap” and an anonymous donor through the AMC foundation.

## Conflict of Interest

The authors declare that the research was conducted in the absence of any commercial or financial relationships that could be construed as a potential conflict of interest.

## Publisher's Note

All claims expressed in this article are solely those of the authors and do not necessarily represent those of their affiliated organizations, or those of the publisher, the editors and the reviewers. Any product that may be evaluated in this article, or claim that may be made by its manufacturer, is not guaranteed or endorsed by the publisher.

## Abbreviations

AHA: American Heart Association
AV: atrioventricular
BSA: body surface area
CAA: coronary artery aneurysm
CABG: coronary artery bypass grafting
CAG: coronary artery angiography
cCTA: coronary computed tomographic angiography
CMR: cardiac magnetic resonance imaging
Cx: circumflex
IVIG: intravenous immunoglobulin
JCS: Japanese Circulation Society
KD: Kawasaki disease
LAD: left anterior descending coronary artery
LMCA: left main coronary artery
LMP: luminal myofibroblastic process proliferation
PTCA: percutaneous transluminal coronary angioplasty
RCA: right coronary artery.
